# Development and application of a 3D periodontal in vitro model for the evaluation of fibrillar biomaterials

**DOI:** 10.1186/s12903-020-01124-4

**Published:** 2020-05-19

**Authors:** Franziska Koch, Nina Meyer, Silvio Valdec, Ronald E. Jung, Stephanie H. Mathes

**Affiliations:** 1 University of Applied Sciences and Arts Northwestern Switzerland, School of Life Sciences, Institute for Chemistry and Bioanalytics, 4132 Muttenz, Switzerland; 2 Department for Chemistry and Biotechnology, Tissue Engineering, Zurich University of Applied Sciences, 8820 Wädenswil, Switzerland; 3 Clinic of Cranio-Maxillofacial and Oral Surgery, Center of Dental Medicine, University of Zurich, University Hospital Zurich, 8032 Zürich, Switzerland; 4 Clinic of Fixed and Removable Prosthodontics and Dental, Material Science, Center of Dental Medicine, 8032 Zürich, Switzerland

**Keywords:** 3D model, Periodontal ligament, Regeneration, Self-assembling peptide

## Abstract

**Background:**

Periodontitis is a chronic inflammation of the tooth supporting structures that finally can lead to tooth loss. As chronic periodontitis is associated with systemic diseases multiple approaches have been followed to support regeneration of the destructed tissue. But very few materials are actually used in the clinic. A new and promising group of biomaterials with advantageous biomechanical properties that have the ability to support periodontal regeneration are self-assembling peptides (SAP). However, there is still a lack of 3D periodontal models that can evaluate the migration potential of such novel materials.

**Methods:**

All experiments were performed with primary human periodontal ligament fibroblasts (HPLF). Migration capacity was assessed in a three-dimensional model of the human periodontal ligament by measuring the migration distance of viable cells on coated (Enamel Matrix Protein (EMP), P11–4, collagen I) or uncoated human dentin. Cellular metabolic activity on P11–4 hydrogels was assessed by a metabolic activity assay. Deposition of ECM molecules in a P11–4 hydrogel was visualized by immunostaining of collagen I and III and fibrillin I.

**Results:**

The 3D periodontal model was feasible to show the positive effect of EMP for periodontal regeneration. Subsequently, self-assembling peptide P11–4 was used to evaluate its capacity to support regenerative processes in the 3D periodontal model. HPLF coverage of the dentin surface coated with P11–4 increased significantly over time, even though delayed compared to EMP. Cell viability increased and inclusion of ECM proteins into the biomaterial was shown.

**Conclusion:**

The presented results indicate that the 3D periodontal model is feasible to assess periodontal defect coverage and that P11–4 serves as an efficient supporter of regenerative processes in the periodontal ligament.

**Clinical relevance:**

The establishment of building-block synthetic polymers offers new opportunities for clinical application in dentistry. Self-assembling peptides represent a new generation of biomaterials as they are able to respond dynamically to the changing environment of the biological surrounding. Especially in the context of peri-implant disease prevention and treatment they enable the implementation of new concepts.

## Background

The periodontium is a highly complex structure within the oral cavity that consists of the alveolar bone, the cementum – attached to the dentin- and periodontal ligament. In case of periodontitis, destruction of the periodontium occurs due to biofilm establishment and subsequent chronic inflammation of the periodontal ligament [1, 2]. If the inflammation in tissue persists, not only the sophisticated soft tissue structure of the periodontal ligament, but also the alveolar bone mass decreases and the cementum is resorbed [3]. In the last stage of periodontitis, the periodontium is no longer able to stabilize the tooth. Periodontitis has shown to be associated to systemic diseases such as cardiovascular disease, diabetes, breast cancer and adverse pregnancy outcomes [4–6], increasing the severity of this periodontal disease and the necessity to address new therapeutic approaches for curing.

Different kinds of treatment strategies to recover the defect of periodontal ligament were established within the last decade. The golden standard is Enamel Matrix Protein (EMP) which is applied as a coating of the dentin roots [7]. Other treatment strategies include the insertion of grafting material to achieve periodontal regeneration [8]. Until today different soft and hard tissue replacement grafts, membranes for guided tissue repair, root surface bio modifications, and delivery of growth factors have been developed [9]. However, the overall success of the intervention therapy highly relies on the surgical skills and experience of the surgeon [10]. Especially ensuring a consistent interface of tooth structures and the material is a critical factor concerning the result. Therefore more emphasis is put on the establishment of regenerative solutions and the development of polymeric biomaterials, which do not primarily support the mechanical stability of the defect pocket, but have the capability to dynamically fulfill the requirements of a changing environment in the re-modulation of the periodontal tissue. Interesting candidates of adaptable systems are small peptides, able to assemble to higher molecular structures under certain conditions, building up a supramolecular nanostructured architecture [11–15]. Self-assembling peptides combine different advantages: For the generation of the peptide based hydrogels, no toxic crosslinkers have to be implemented, the peptides are highly biocompatible and degradation products can be metabolized by the cells. As a consequence, these hydrogel scaffolds are emerging to attractive alternatives for tissue engineering scaffolds, exhibiting a biomimetic environment with a spatial and temporal regulation [16]. Multiple approaches aiming at the implementation of these peptide based scaffolds exist for the regeneration of different tissues [17]. One of these peptide classes have been developed by the group of Aggeli et al, termed as P11-peptides [18]. These peptides consist of 11 amino acids and are able to form antiparallel β-sheet structures under defined conditions. Based on the favorable thermodynamic energy these β-sheet structures will form nanotapes via hydrogen bonding [19, 20]. Nanotapes subsequently stack to form ribbons, which are stabilized by π – π interactions of the aromatic residues in the motif. The hydrogel character is formed by aggregation of peptide ribbons to give fibrils that aggregate to form 3D fiber networks and entraps water. One of these peptides, a 11-mer peptide P 11–4 (Ac-QQRFEWEFEQQ-NH_2_) has been investigated as an injectable scaffold for treating bone defects, or is used for treatment of dental hypersensitivity or decays [21, 22].

Beside the development of new polymeric regenerative solutions such as the SAP P11–4, there is still an unmet need of robust and predictable in vitro models that imitate the highly complex structure of the periodontium. Generally, in vitro systems are well accepted especially in cases where a pre-evaluation of multiple possibly advantageous candidates is mandatory. The most commonly used systems are performed in standard cell culture plates and lack the natural surface of the tooth root surface [23]. Moreover, they do not take into account that the periodontal fibroblasts of the ligament have to be encouraged to migrate out of a three dimensional environment into an offered endogenous matrix. Most of the systems evaluate the effect (e.g. cell viability, proliferation, gene expression, migration) of soluble factors on a cell population present on a plastic surface [24–26]. But in order to enable a more realistic view and to get closer to the conditions that are displaced in vivo, three-dimensionality and tooth root surface should be taken into account.

Hence, the development of an adequate in vitro model and its application for assessing the regenerative potential of P11–4 are presented in this article. Although the periodontal ligament possess a large reservoir of different cell types such as fibroblasts, epithelial cells, osteoblasts, cementoblasts, and mesenchymal stem cells, human periodontal ligament cells were chosen as cell model, as they will be the first cells that come in contact with a injected biomaterial [27]. In order to still meet the prerequisite of robustness and reproducibility, key parameters for periodontal regeneration were defined: I) significant increase of viability of human primary periodontal ligament fibroblasts II) migration of these cells on and into a polymeric matrix and III) capability of ECM protein deposition. In the course of in vivo tissue regeneration increased expression of tissue specific ECM [28] and the establishment of ligament structures is aspired. In the case of the periodontal ligament, it is mainly collagen type I [29] and in lesser amount collagen type III, IV, V, VI and XII [30]. Next to collagen also oxytalan fibers contribute to the specific biomechanical behavior of the periodontal ligament [31].

Therefore, the aim of the present study was the development of a test system, including a 3D periodontal model that can predict the success of novel polymeric biomaterials. Further, we address the question, if SAP (P11–4) as polymeric biomaterials would serve also as an appropriate matrix to promote the regeneration of the periodontal ligament.

## Methods

### Cell culturing

For all experiments human periodontal fibroblasts (HPLF; purchased from ScienCell, #2630) until passage 6 were used. Cells were cultivated at 37 °C and 5% CO_2_ in Dulbecco’s Modified Eagle’s Medium (DMEM) - high glucose medium (Sigma; D6429; with Penicillin-Streptomycin (P/S 1%; Sigma- Aldrich; P0781; USA) and fetal bovine serum (FBS 10%; Sigma Aldrich; USA).

### Harvest human dentin

Dentin pads were produced from out of the crowns of freshly extracted and caries free human molars. The indication for tooth extraction was periodontitis in most of the cases. Patients were informed on the further usage of the teeth and signed an informed consent for anonymous in vitro analysis.

Raw blocks, enamel and pulp free, were cut using a precision diamond wire saw (Model 3241 Precision Vertical Diamond Wire Saw; Well Diamond Wire Saws, Inc., Georgia, USA).

The blocks were then polished to a volume of 7x4x1mm (LaboPol-21, Type No. 529, Struers, Rødovre, Denmark), removing all attached cementum. The dentin pads were stored in 75% ethanol at 4 °C prior to further analysis.

### 3D model of human periodontium

**Fig. 1:**
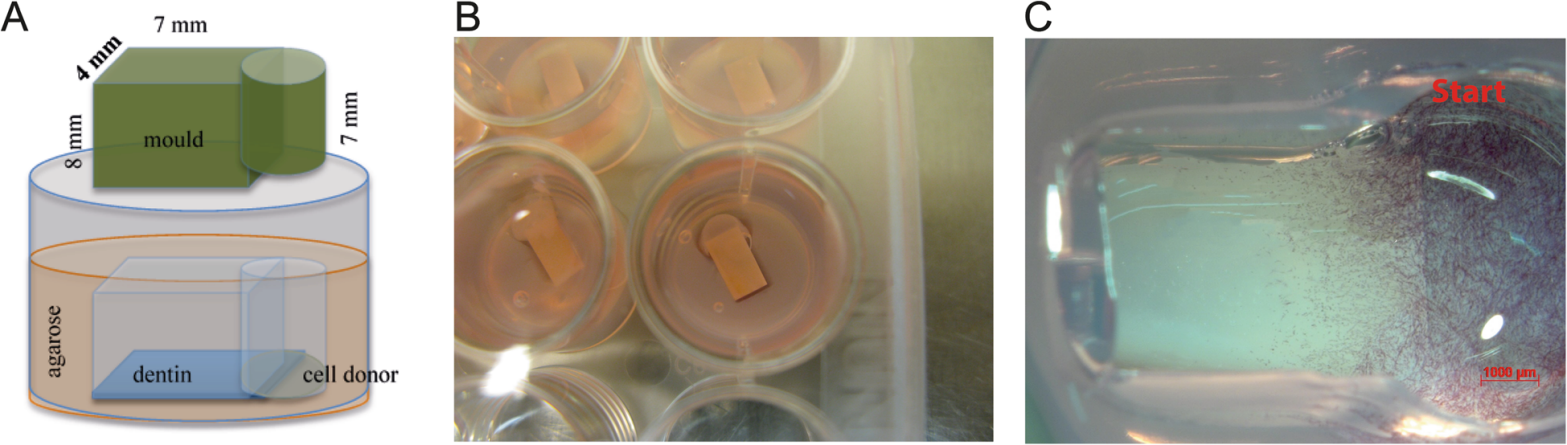
In vitro model set up. **a)** Illustration of the mould embedded in agarose gel (2 mg/ml) and the human dentin plate on the bottom with the collagen based cell donor compartment **b)** Cultivation for 4 and 8 days with exchange of the medium every other day **c)** Cell migration into the applied matrix, after 8 days incubation. Cells were visualized with a metabolic activity reagent (3-(4,5-Dimethylthiazol-2-yl)-2,5-diphenyltetrazolium bromide, MTT)

A novel in vitro model with the ability to imitate a periodontal pocket was developed to assess the potential of polymeric biomaterials for periodontal regeneration. As cell migration capability from the periodontal ligament to an implanted biomaterial is a key parameter to achieve a periodontal regeneration, the 3D in vitro model was designed in a way to study the migration of cells from one 3D compartment to another 3D compartment. In order to come close to the complex periodontal pocket situation, the 3D compartments were build up on human dentin disk, simulating the eroded tooth side. The model consists of two components: a) a 4x7x1 mm piece of human dentin that served as a supportive basis for the peptide and b) HPLF cells embedded in a collagen type I hydrogel to build up a cell donor (Fig. 1).

In order to produce the cell donor gel HPLF were cultivated until the passage 6 in medium and mixed with medium and 2 mg ml^− 1^ collagen type I (Corning® high concentration 8.38 mg/ml in 0.02 N Acetic Acid, Rat Tail) to reach a cell concentration of 1.3*10^4^ cells ml^− 1^. 150 μl of the mix was pipette in the 96 well plates and cultivated at 37 °C and 5% CO_2_ for 2–3 days.

To avoid the migration of the HPLF cells over the edges and to the cell culture plate, a surrounding matrix was formed with agarose Type II-A (Sigma A9918; Spain, 2 mg ml^− 1^). Agarose was heated up to 80 °C and 300 μl were pipetted to the wells of a 48 well plate. After solidification, self-made moulds were introduced to keep the space for the upcoming insertion of the dentin pad and collagen hydrogel. Moulds were deposited aligned in the same direction on the agarose bottom and were filled with another 350 μl of agarose (80 °C) and removed carefully after the agarose hardened.

The dentin matrix was put in the square part of the hole. Subsequently, the collagen cell donor compartment was carefully harvested out of the 96 well plates and placed in the receiving compartment of the form so 50% of the gel pad covered the dentin and the other 50% laid on the agarose (covered with a drop of collagen hydrogel for better attachment). The wells were carefully filled with 1 ml pre-warmed medium and incubated for 4 and 8 days. Every other day the medium was changed.

### Cell migration evaluated in 3D periodontal model

To study HPLF migration on the different test substances, the medium was carefully removed and Thiazolyl Blue Tetrazolium Bromide (MTT; Sigma Aldrich) at a final concentration of 1 mg/ml solution in culture medium was added and incubated for 2 h. Viable cells were detected with a binocular STEMI SR (Zeiss; 0.8x; Germany) by the intracellular reduction of MTT into violet coloured formazan. Determination of cell migration and density was assessed with ImageJ software (v1.52i). Cell migration distance was measured for the farthest migrated cells and the area, were most of the cells have been migrated.

### Preparation of self-assembling peptide (SAP)

Peptide hydrogels were freshly prepared prior to the respective experiment. The lyophilized peptide P11–4 (CH_2_-Ac-Gln-Gln-Arg-Phe-Glu-Trp-Glu-Phe-Glu-Gln-Gln-NH_2_, peptide content 95%, ammonium salt) was purchased from Biosynth AG (Staad, Switzerland). Quality control was done by HPLC and mass spectroscopy (MS) stored at − 20 °C prior use. Working concentration was set to 20 mg ml^− 1^ for all experiments. The peptide was first dissolved in sterile Tris buffer (Roche Diagnostics; Germany; 0.055 M; pH 8) resulting in a monomeric peptide solution with a concentration of 40 mg/ml. To achieve final peptide concentration of 20 mg/ml and to induce peptide self-assembling, an equal volume of neutralizing buffer (0.055 M Tris, 0.192 M NaCl, pH 7.0) was added to peptide monomer solution. This procedure resulted in the formation of a translucent hydrogel with pH 7.4. Sterilization of the peptide gel was done by exposure to UV light 20 min 20 W at 365 nm.

### Investigation of dentin / peptide interaction

Whenever a material is going to be evaluated by means of the periodontal model, the interaction of the material with the underlying natural matrix (the dentin) has to be considered in order to obtain information regarding the stability of the interface. In order to investigate the penetration capacity of the peptide gel into dentin channels, a Fluorescein-βA-12-Q-NH2 labeled peptide (ATTO647-P11–4; JPT Peptide Technologies GmbH) was used in a mix of 1:10 with unlabeled P11–4 in a concentration of 20 mg/ml. Prior to application of the material onto the dentin pads, demineralization of the tooth material was performed to enable subsequent cryo-sectioning. To achieve this, the pads were treated with 6% acetic acid in the ultrasound bath and stored in 5% Ethylenediaminetetraacetic acid EDTA (Sigma-Aldrich; CAS 60–00-4) in water, for 2 days at 4 °C.

After 24 h incubation of the fluorescent labeled peptides, cryo-sections were taken. Samples were shock frozen with liquid nitrogen and embedded in Tissue-Tek® O.C.T. Compound, (Sakura® Finete, USA) cut in the direction from the dentin towards the P11–4 with MICROM HM 550 (Histocom AG; Germany) resulting in 10 μm slides. The pictures were taken with Olympus fluorescence microscope (IX81Motorized inverted research Microscope, with U-MWIGA3 Olympus; Emission filter BP575–625).

### Determination of metabolic activity

For the assessment of the peptide hydrogel biocompatibility, HPLF (4000/well) were seeded on P11–4 hydrogels and cultured up to 6 days. As a positive control 2 mg ml^− 1^ collagen type І hydrogel (Corning® high concentration 8.38 mg ml^− 1^ in 0.02 N Acetic Acid, Rat 354,249; USA.) was prepared with medium. After 1, 3 and 6 days incubation, Prestoblue® (Cell Viability Reagent, Invitrogen, A13262; USA) assay was performed, according to the manufacturer protocol. Finally, the metabolic activity was quantified by measuring highly fluorescent resorufin that is produced by the intracellular reduction of the PrestoBlue® Reagent. The obtained fluorescence signal was measured with FLUOstar OPTImA (BMG LABTECH; Germany; Excitation filter 550–10; Emission filter 590–10). Cells cultured on TCPS after 1 day served as control and were set to 100%. The experiment was performed in triplicates.

### Immunostaining of ECM components

As not only cell migration and increase of cell number in a defect site, but also the establishment of a native ECM is essential for successful tissue regeneration, the deposition of important ECM molecules such as collagen I, III and fibrillin I of the periodontal ligament was analyzed. HPLF cells were seeded on P 11–4, 2000 cells/well and cultured up to 7 days in a 96 well plate.

After 7 days incubation, human collagen I and IIІ as well as fibrillin I were detected by fluorescent immunostaining. The cells and the proteins were fixed with formalin (10%, Sigma Aldrich; USA) for 10 min, blocked with 1% bovine serum albumin (Sigma- Aldrich; USA) and permeabilized using 0.1% Triton-x-100 (Fluka; in Dulbecco’s Phosphate Buffered Saline (DPBS; Sigma- Aldrich;D8537; United Kingdom)). As a next step, primary antibodies (purchased from Abcam, Cambridge, UK) against human collagen I (ab34710,rabbit polyclonal to collagen I) and collagen III (ab6310, mouse monoclonal to collagen III) as well as human fibrillin I (ab53076, rabbit polyclonal to fibrillin I) were prepared at 1:500 in blocking solution and incubated for 1 h at room temperature. Afterwards samples were washed three times with PBS (5 min). Finally, the secondary antibody (goat anti mouse IgG (H + L), Invitrogen A11031¸ Alexa Fluor 568; USA) was diluted 1:400 in blocking solution and incubated for 2 h in the dark. To visualize cell nuclei, cells were counterstained with DAPI (Sigma AldrichD8417-5MG) for 10 min in the dark. Prior microscopy, samples were washed three times with PBS. Pictures were taken with a IX81 motorized inverted research microscope with the filter U-MWU2 (Olympus; DAPI Excitation filter 330–385 and Emission filter 420 nm; TRITC Excitation filter 530–550 and Emission filter 570 nm; USA).

### Quantification of collagen I expression

HPDLF were cultured up to 21 days at a density of 2000 cells/cm^2^ in a 96 well plate on top of P11–4 hydrogels, prepared at 20 mg/ml. During the culturing time, the medium was changed every three days. Pro-collagen type І expression was analysed in cell culture supernatant at day 7, 14 and 21 using the MicroVue CICP procollagen detection kit (TecoMedical AG, Sissach, Switzerland) per manufacturer’s protocol.

### Cell migration into peptide matrix using confocal laser scanning microscopy (CLSM)

To study cell migration, HPLF (2000 per well) were seeded on top of P11–4 (20 mg/ml) peptide hydrogels were prepared in a 96 well plate. After 4 days of incubation the actin cytoskeleton of the cells were stained with Alexa Fluor 568 phalloidin (Invitrogen, A12380; USA). The picture was taken with the confocal microscope (FV 1000; Olympus, Laser 559 nm).

### Statistical analysis

All data were analyzed with GraphPad Prism version 7.0. Statistical significance was determined by performing either a one-way or a two-way ANOVA test, followed by Tukey multiple comparison posthoc test. *P*-values ≤0.05 were considered as significant. Cell experiments were done with HPDLF from three independent donors and with three technical replicates per run. The investigation of peptide-dentin interaction was performed in three technical replicates. All data, represent the mean values ± standard deviation.

## Results

### Set-up of the 3D periodontal in vitro model (proof-of-concept)

**Fig. 2:**
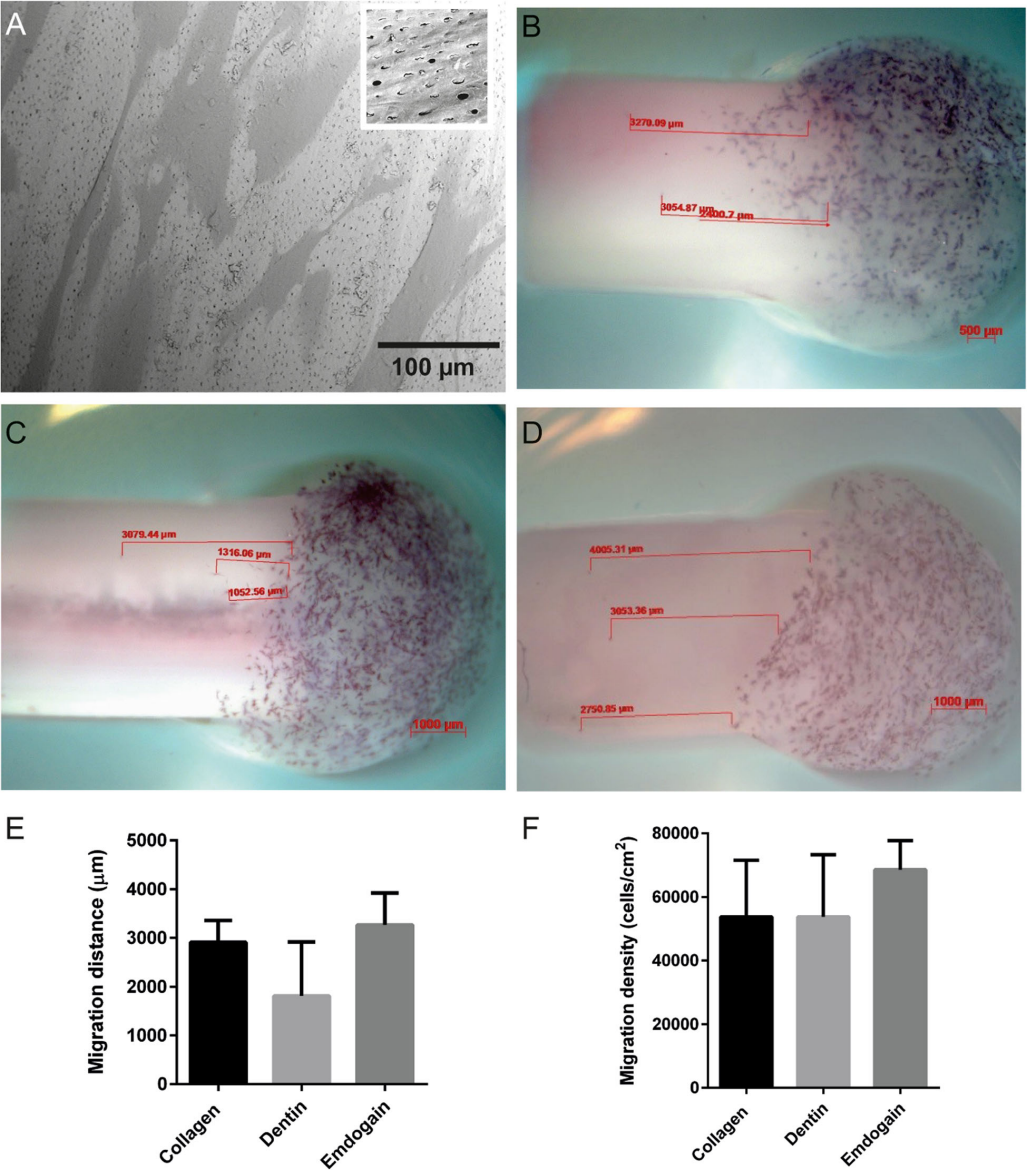
Proof of concept study. The interaction of human periodontal ligament fibroblasts (HPLF) with the different components used in the developed 3D in vitro model was first assessed by Scanning Electron Microscopy (SEM) and cell staining with MTT. **a**) SEM of HPLF grown on human dentin (scale bar 100 μm). Migration of HPLF was visualized by cell staining with MTT after 4 days of culture on cultured on **b**) collagen (2 mg/ml, scale bar 800 μm), **c**) dentin (scale bar 1000 μm) and **d**) Enamel Matrix Protein (EMP, 30 mg/ml, scale bar 1000 μm). **e**) Quantification of the maximal migration distance (μm) of HPLF on collagen, dentin and EMP (30 mg/ml) was determined by ImageJ analysis, *n* = 3. Data showed no statistical difference. **f**) Migration density calculated as cell number/cm^2^) with ImageJ analysis

As proof of concept of the developed 3D in vitro model, migration of human periodontal ligament fibroblasts (HPLF) was assessed on human dentin and dentin covered with either collagen I and or EMP (Fig. 2). The distance of migrated cells out of the donor hydrogel was assessed after 4 days. HPLF cells were able to migrate in all conditions. An approximately two fold lower migration distance (μm), although not statistically significant, was found for HPLF cultured on non-coated human dentin in comparison to collagen or EMP coated human dentin. However comparable migration density (number/cm^2^) was detected for coated and non-coated human dentin.

### Investigation of SAP using periodontal model

#### Interaction of SAP and human dentin

**Fig. 3:**
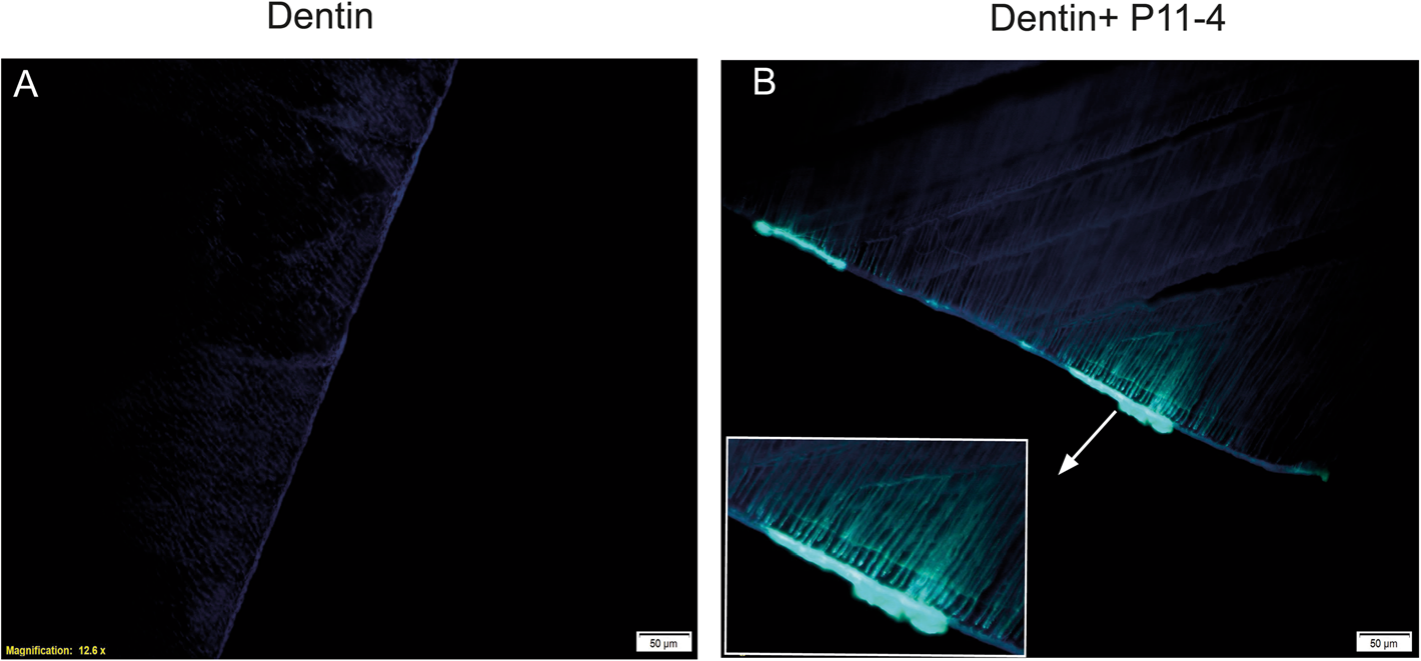
Time dependent penetration of P11–4 into dentin tubules. **a**) Confocal microscopy of human dentin plate (scale bar 50 μm). **b**) Penetration of labeled self-assembling peptide P11–4 (1:10 mix with a 20 mg/ml concentration) in dentin tubuli was tracked by confocal microscopy after 4 days incubation (scale bar 50 μm). A deep penetration of P11–4 into dentin tubuli can be observed

To study the interaction of fluorescence labeled P11–4, as an example of a polymeric test substance, with human dentin, an immunohistochemical evaluation was performed (Fig. 3). After 4 days incubation, P11–4 (prepared as monomeric solution) interacts with the human dentin surface and penetrates deeply into the dentin tubules (Fig. 3b).

#### Metabolic activity of HPLF on SAP hydrogels

**Fig. 4:**
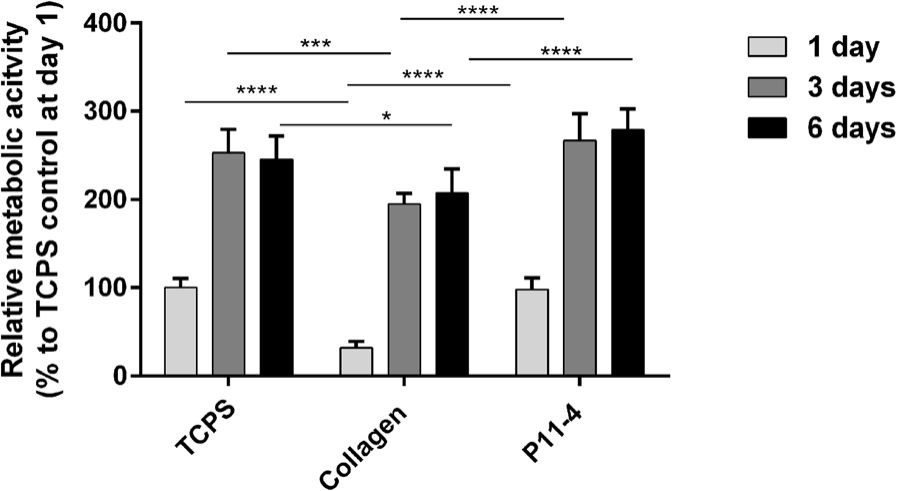
Metabolic activity of HPLF. Cell viability was measured after 1, 3 and 6 days incubation on tissue culture polystyrene (TCPS), collagen (2 mg/ml), P11–4 (20 mg/ml) using a metabolic activity assay. Data were calculated to % TCPS control at day 1, *n* = 3, * *p*-value ≤0.05, *** *p*-value ≤0.001, **** *p*-value ≤0.001. HPLF cultured on P11–4 showed a significant increase in metabolic activity, compared to cells grown on collagen gels

In order to ascertain the biocompatibility of the peptide hydrogel, the metabolic activity of HPLF was evaluated in advance on P11–4 hydrogels over a period of 6 days. In general, an increase in metabolic activity can be observed in a time dependent manner indicating cellular growth (Fig. 4). HPLF seeded on collagen hydrogels for one day showed a 50% reduced metabolic activity in comparison to cells cultured on TCPS and P11–4 hydrogels. A similar pattern in metabolic activity has also been observed after 3 and 6 days incubation.

#### Cell migration into peptide matrix

**Fig. 5:**
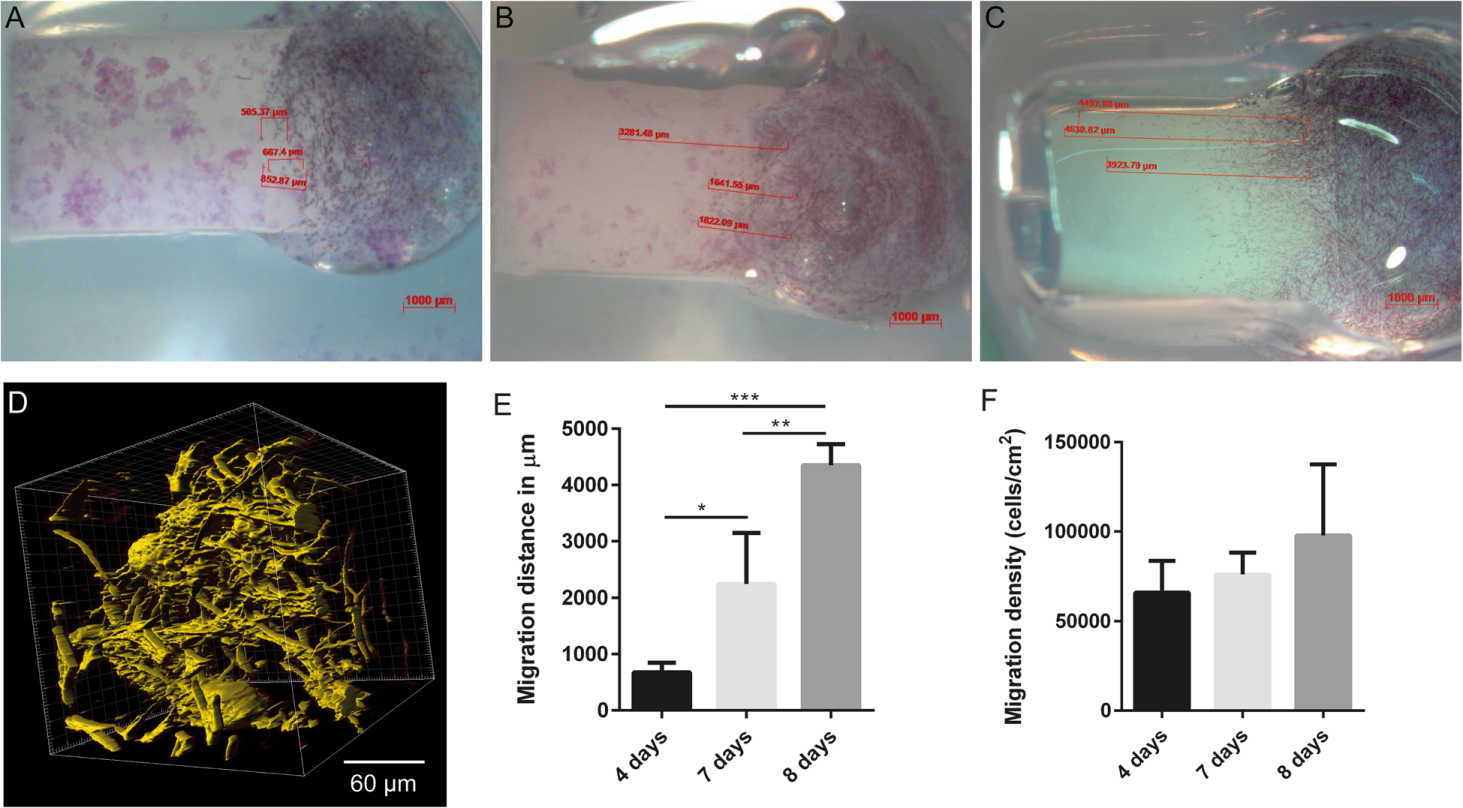
HPLF migration on P11–4 peptide hydrogel. (**a**) Cell migration was measured by MTT staining after 4 days, 7 days and 8 days culturing on P11–4 hydrogels (20 mg/ml). **b**) 3D Z-stack image of migrated HPLF on P11–4 hydrogel after 3 days, taken by confocal microscopy. Scale bar 60 μm. (**c**) Migration distance (μm) was measured with Axiovision software (Zeiss) from baseline after 4, 7 and 8 days incubation of HPLF on P11–4 (20 mg/ml), n = 3, *p-value ≤0.05, **p-value ≤0.01, *** *p*-value ≤0.001. (**d**) Cell migration density in number/cm^2^ after 4 and 8 days incubation of HPDLF. **e**) Quantification of the maximal migration distance (μm) of HPLF on P11–4 (20 mg/ml) was determined by ImageJ analysis, n = 3, *p-value ≤0.05, **p-value ≤0.01, *** *p*-value ≤0.001. **f**) Migration density calculated as cell number/cm^2^ with ImageJ analysis. Pictures proof a high migration capacity of HPLF into fibrillar P11–4 hydrogels

The migration of HPLF cells in a fibrillar peptide hydrogel, such as P11–4, was analyzed in respect to the cellular distribution and cell morphology in and on the peptide matrix. By phalloidin actin staining it was shown that the cells obtained a stretched phenotype and were homogeneously distributed throughout the synthetic matrix (Fig. 5). The migration distance increased significantly in a time-dependent manner. Within 4 days of culture (time point 1 to time point 3), cells were able to migrate a distance of 3700 μm (Fig. 5e). The migration density were found to increase also in a time-dependent manner, however not statistically significant (Fig. 5f).

#### Matrix protein deposition

**Fig. 6:**
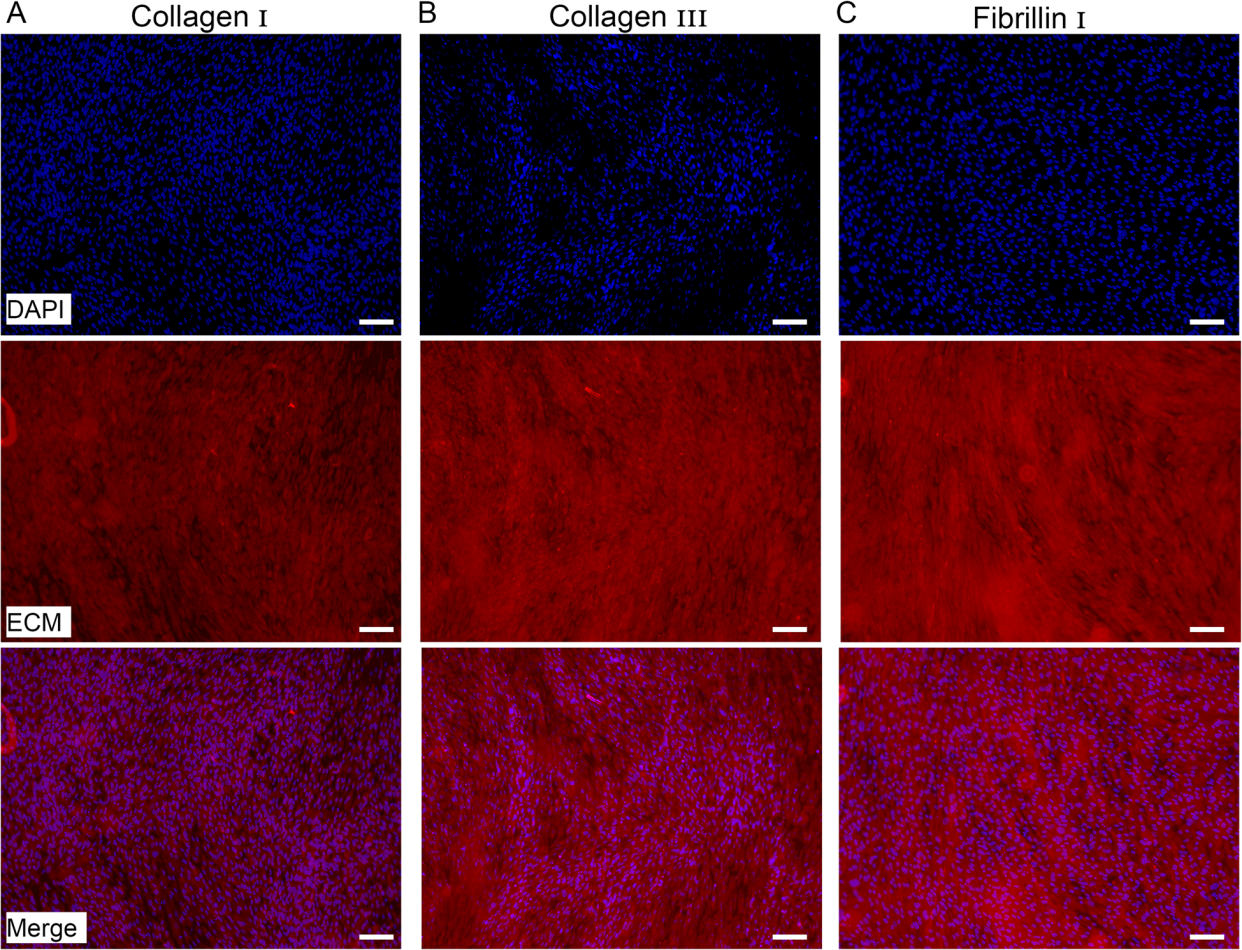
Extracelluar matrix (ECM) protein expression. **a**) Collagen type I, **b**) Collagen type III and **c**) Fibrillin I was visualized with fluorescent immunostaining after 7 days incubation on P11–4 (20 mg/ml) hydrogels. Scale bar 100 μm. Pictures demonstrate the expression of relevant ECM proteins for periodontal regeneration, after the incubation of HPLF on P11–4 hydrogels

The issue that was addressed in this experiment was the question of the capability of the peptide matrix to allow ECM molecule deposition (collagen I, III and fibrillin I; Fig. 6). Confocal microscopy pictures demonstrated the deposition of collagen I, III and fibrillin matrix around the cells that were cultured within P11–4 peptide matrices.

**Fig. 7:**
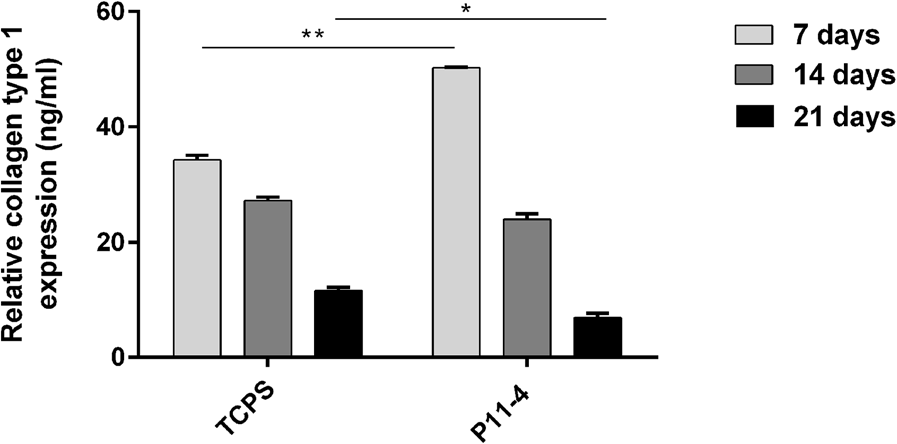
HPLF collagen type Ι expression. HPLF were cultured on P11–4 hydrogels (20 mg/ml) up to 21 days. After 7, 14 and 21 days, supernatants were removed and analyzed for collagen type Ι expression by ELISA. Collagen amount were normalized to the cell proliferation rate. As a control, cells were cultured on tissue culture polystyrene (TCPS). Data represent the mean ± standard deviation, n = 3, *p-value ≤0.05, **p-value ≤0.01. Cells cultured on P11–4 hydrogels resulted in a significant increase in collagen type Ι production, compared to HPLF grown on TCPS

To confirm the results of extracellular matrix production observed by confocal microscopy, the expression of the most important extracellular matrix protein (collagen type Ι) was assessed also quantitatively (Fig. 7).. After 7 days of culture of HPLF on P11–4 hydrogels, collagen type Ι expression was statistically significant increased by 1.5 fold compared to TCPS control. However after 21 days, the collagen amount in the supernatants of HPLF was approximately 2 fold lower on P11–4 peptide matrix samples compared to TCPS control samples.

## Discussion

The presented study aimed at the establishment of a robust and predictable in vitro test system for the evaluation of polymeric biomaterials designed for periodontal regeneration. To test the feasibility of the newly developed 3D in vitro model, the periodontal regenerative capability of the synthetic self-assembling peptide P11–4 was assessed in a subsequent experiment.

In vitro systems using human primary cells are commonly used in order to gain evidence of action and hazard of molecules and biomaterials. This is also true for the modulation of cellular processes within the human periodontium. Weinreb and Nemcovski summarized existing 2D and 3D in vitro models established for the evaluation of periodontal wound healing [23]. They highlighted different aspects of periodontal wound healing which are simulated in according cellular models. In most of the published studies cell behavior in a two-dimensional (2D) environment was investigated. Only few approaches address the natural three-dimensional environment. But it is a crucial fact that cells are exposed to a 3D environment in vivo and have to be provoked to fill an existing gap and to establish neo-tissue structures.

Therefore, the aim was to establish a 3D periodontal evaluation model, encompassing the native supporting structure of human dentin and a cell homing compartment, resembling surrounding tissues. A collagen gel served as a scaffold where human primary periodontal fibroblasts were embedded. Primary outcome of our periodontal screening model was the migratory capability of the cells from out of the cell-donor gel in order to cover efficiently the disclosed root structure. This parameter was prioritized in respect to proliferation or differentiation as in the course of defect healing the critical issue represents the time until defect coverage and has to oppose the microorganisms’ invasion. By evaluating the described parameters above, the regenerative potential of a polymeric biomaterial can be assessed in 3D.

In order to achieve an acceptable balance between robustness and complexity one cell type was included in this first approach. Essential structures like the dentin surface and surrounding collagen embedded periodontal fibroblasts were represented within the model. Hence, multi-well assays could be set up in less than 3 min, assuming that the collagen gels and dentin pads are prepared. Non-destructive model analysis is challenging due to intransparency of the dentin pads. Immunofluorescent or- histochemical methods are not applicable due to a) high autofluorescence of collagen structures of the prepared dentin and b) insufficient penetration of antibodies through peptide hydrogel. Further analytical procedures like focused ion beam/scanning electron microscope tomography [32] or mirco-CT analysis [33] have to be evaluated in order to characterize the model and established nanostructures in more detail.

One of the most extensively investigated biomaterial used for the regeneration of the periodontal ligament -in vitro as well as in vivo- is enamel matrix derivative, applied as a formulation known as EMP.

Besides 2D, also 3D models were used to investigate the mechanisms and feasibility of the porcine enamel proteins. In these models, relevant cells (i.e. osteoblasts and periodontal ligament cells) were seeded onto bone-substitute particles [34] grown within collagen gels [35] or seeded as a three-dimensional (organoid) construct onto a rigid support [36, 37] and allowed to grow and mature. But it has to be considered that the disclosure of root surface structures occurs in the periodontal pocket in the case of periodontitis. Hence, any material, inserted into the pocket has to exhibit the feasibility to interact with the exposed dentin. Davenport et al. [38] could show that application of enamel matrix proteins supported attachment of primary human fibroblasts to diseased dentin structures. But still, the integration of a native dentin structure into a three-dimensional evaluation system was not described yet. As multiple in vitro studies already proved the efficacy of enamel matrix proteins and due to it’s routine use in clinical processes, it served to show the validity of the newly established 3D periodontal in vitro model. Our model revealed that periodontal fibroblasts migrated slightly faster on EMP covered dentin surface compared to collagen or uncoated ones, as migration distance from out of the donor compartment was higher (3269.5 μm ± 526) compared to both controls (dentin = 1815.6 ± 967 μm, collagen = 2908.5 ± 498 μm). The efficient migration capacity of the periodontal cells on pure dentin surface does nor occur unexpectedly, as the model lacks the microbial component. In order to reveal if the observed enhanced migratory capacity of the cells is due to the migration of some single cells or if the cells travel in coherent groups, images were analyzed using another algorithm. When images were evaluated concerning the denseness of migrating cells, no difference was found between the groups. In others words, EMP promoted the migration of some single cells, but not of a crucial cohort of cells, needed for gap filling. In the literature contradictory conclusions in respect to the impact of enamel matrix proteins on cell migration can be found. While Kasaj et al. [39] concluded that soluble EMD significantly increased migration properties, Chong et al. [40] demonstrated that EMD supports would filling only to a certain extend. It has to be emphasized that in the presented study unlike in most published studies, EMD was not used as a soluble additives, but as in the clinical routine as EMP formulation. Nevertheless, our results support previous studies, by showing the positive impact of migration capacity of PDLF cells, but limited wound filling properties.

In contrast enamel matrix proteins which act primarily via soluble protein debris from nanospheres [41] the material of interest in the presented study (self-assembling peptide hydrogel) acts as a scaffold, providing a structure for cell embedding and absorption of proteins either secreted from the cells or present in surrounding fluids. Koch et al. investigated physicochemical properties of a variety of the P11-x family [42] and showed the cytocompatibility of two single-peptide systems, namely P11–4 and P11–8 as suitable candidates for the support of regenerative processes, as the hydrogels of these peptides allow adsorption of fibronectin, attachment of PDLF and human osteoblasts as well as metabolic activity of cells on peptide hydrogel [43]. Like in our results no statistical difference was detected in respect to TCP control after 24 h. We choose P11–4 as a test compound of the 3D PDL system as this peptide already proofed beneficial impact in respect to biomineralization of enamel tooth structures [22, 44].

We could show that P11–4 thoroughly interacts with human dentin and diffuses in dentin channels, thus providing an efficient anchorage to the underlying native material. Although P11–4 peptides do not contain any cells attachment sites, the periodontal fibroblasts were able to elongate and spread within the peptide hydrogel. This can be explained by the observation that attachment proteins like fibronectin adsorb on the surface and presumably also inside the gel, allowing establishment for adhesion points [43]. Using the in vitro model of the periodontal ligament, it was shown that human periodontal ligament cells were capable of migrating and colonizing P11–4 coated dentin. Compared to respective values for collagen and Emdogain®EMP where cells reached a distance of 300–4000 µm after 4 days, cells covered a distance of 1000 µm on P11–4. But distance and denseness of migrating cells increase over time, which may also be attributed to cell proliferation.

Besides peptides of the P11-x family other short peptides able to form solid structures by self-assembling for regenerative purposes gained more attention [17, 45].

The most intensively investigated self-assembling peptide for tissue engineering and regenerative purposes is RADA 16, which consists of 16 alternating hydrophobic and hydrophilic amino acids. Like P11–4 RADA 16 forms a stable β-sheet structure and self-assembles into nanofibers to produce a scaffold hydrogel [46]. Bradshaw et al. investigated the migratory capacity of skin cells (keratinocytes and fibroblasts) on pure RADA 16 and two variants with functional motives. In relation to the observations on RADA16 only the collagen I motive variant could significantly increase cell migration [47]. Comparably to the results of the presented study, unmodified peptide hydrogel does not impede cell migration, but due to the absence of adhesion motives, cell adhesion and migration can only occur as soon as serum proteins attach to the fibril network. In the case of P11–4 not only fibronectin will adsorb the hydrogel matrix [43], but also endogenous produced ECM proteins like collagen I, III and fibrillin I as the qualitative analysis showed. This indicates the likely integration of main periodontal ECM proteins which are important for functional tissue repair. Quantitative analysis of the most prominent ECM protein collagen Ia1 revealed a significantly increased expression after 7 days for P11–4 coated compared to cells grown on uncoated cell culture plates. Under both conditions a down-regulation of collagen Ia1 expression was detected for later time points. This fact may be attributed to the circumstance that the cells are cultivated under static instead of dynamic conditions. Differentiation processes and production of ECM specific proteins of human periodontal ligament fibroblasts depend significantly upon mechanical stimulation [48, 49].

In the case of RADA 16 in vitro studies with human periodontal fibroblasts indicated successful adsorption of collagen I and III which was significantly increased by functionalization of the monomers [50]. Nevertheless unmodified RADA 16 was able to accelerate bone formation during the early healing phase of surgically created periodontal defects in rats [51]. In contrast to unfilled defects, periodontal ligament- like collagen bundles established in the RADA 16 treated group after 4 weeks healing. So it remains questionable if functionalization at all will provide an advantage in vivo, as other factors may cover the functional groups before cells get in contact with the material. With respect to this finding and as P11–4 itself exhibits the potential of protein adsorption, a functionalization seems not necessary for a successful performance. But is still remains to be proven in an in vivo experimental set-up, if the synthetic hydrogel will support and enable periodontal tissue regeneration as first hints of the 3D periodontal model let suspect.

## Conclusion

Relevant and predictable in vitro models are getting more and more important to describe and evaluate biomaterials for tissue regeneration. Not least driven by authorities that request more information concerning the safety, risk-benefit and mechanistic behavior of implanted materials. For the evaluation of biomaterials and particularly of polymeric ones we developed a test system that includes human native dentin as the support structure for the subject of interest. Using this approach the colonization of the gap with tissue restoring cells can be evaluated by the parameters of migration speed and denseness of migrating cells. A new candidate for periodontal regeneration, the hydrogel formed by P11–4 monomers was evaluated with the means of this 3D system. The results let to the conclusion that this synthetic smart biomaterial which is able to respond to shifts of pH, temperature and ionic strength seems to be adequate to promote the tissue regeneration process. In order to still meet the prerequisite of robustness and reproducibility, key parameters for periodontal regeneration were defined: I) significant increase of viability of human primary periodontal ligament fibroblasts II) migration of these cells on and into a polymeric matrix and III) capability of ECM protein deposition. In the course of in vivo tissue regeneration increased expression of tissue specific ECM. Currently, P11–4 hydrogels are evaluated in an ongoing animal trial in order to validate the periodontal in vitro model.

## Data Availability

The datasets used and/or analysed during the current study are available from the corresponding author on reasonable request.

## Abbreviations

ECM: Extracellular matrix
ELISA: Enzyme linked immunosorbent assay
HPLF: Human periodontal ligament fibroblasts
MTT: Thiazolyl Blue Tetrazolium Bromide
P: Peptide
PDL: Periodontal ligament
SAP: Self-assembling peptide
TCPS: Tissue culture polystyrene
